# Maitjara Wangkanyi: Insights from an Ethnographic Study of Food Practices of Households in Remote Australian Aboriginal Communities

**DOI:** 10.3390/ijerph17218109

**Published:** 2020-11-03

**Authors:** Suzanne Bryce, Inawantji Scales, Lisa-Maree Herron, Britta Wigginton, Meron Lewis, Amanda Lee, Ngaanyatjarra Pitjantjatjara Yankunytjatjara (NPY) Women’s Council

**Affiliations:** 1 Ngaanyatjarra Pitjantjatjara Yankunytjatjara (NPY) Women’s Council, Alice Springs NT 0871, Australia; cfws.ntl@npywc.org.au (S.B.); inawantji@gmail.com (I.S.); 2 School of Public Health, The University of Queensland, Herston QLD 4029, Australia; l.herron@uq.edu.au (L.-M.H.); b.wigginton@uq.edu.au (B.W.); m.lewis@uq.edu.au (M.L.)

**Keywords:** Aboriginal, diet, food insecurity, food choice, ethnographic

## Abstract

Many historical, environmental, socioeconomic, political, commercial, and geographic factors underscore the food insecurity and poor diet-related health experienced by Aboriginal people in Australia. Yet, there has been little exploration of Aboriginal food practices or perspectives on food choice recently. This study, with 13 households in remote communities on the Anangu Pitjantjatjara Yankunytjatjara (APY) Lands, fills this gap using ethnographic and Indigenist methods. Results highlight Anangu resourcefulness, securing food despite poverty and adversity, and provide unique insights into factors influencing the three major types and range of dietary patterns identified. These factors include household economic cycles and budgeting challenges; overcrowding and family structures, mobility and ‘organization’; available food storage, preparation and cooking infrastructure; and familiarity and convenience. Structural and systemic reform, respecting Aboriginal leadership, is required to improve food security.

## 1. Introduction

Prior to colonization of Australia, all available evidence suggests Aboriginal people enjoyed a varied traditional diet and were in good nutritional health [1]. European invasion impacted dramatically on Aboriginal food practices (the multiple activities and customs of food production, gathering/acquisition, preparation, consumption and uses other than for eating). Traditional food sources became increasingly depleted and/or inaccessible, with policies controlling traditional food procurement [2] and population pressure around missions and settlements, forcing Aboriginal people to become dependent on introduced foods, such as flour, tea, and sugar [3,4,5]. This has had adverse consequences for the nutritional health of Aboriginal and Torres Strait Islander people [1,6], who now have high rates of premature death due to preventable diseases, such as cardiovascular disease, type 2 diabetes, some cancers, and kidney disease [7].

Many historical, environmental, socioeconomic, and geographic factors contribute to the current diet of Aboriginal and Torres Strait Islander people. These include sustained economic disadvantage (low incomes and high rates of unemployment), limited educational opportunities, disruption to family structures, overcrowding, high food costs, and inadequate housing, and health hardware such as cooking equipment and food storage facilities [8,9,10,11,12]. Previous research with Aboriginal and Torres Strait Islander communities has found that food choice is affected by availability, affordability, traditional and individual preferences, familiarity, levels of food and nutrition literacy and cooking skills, and cultural factors, such as demand-sharing [1,13,14,15,16,17,18].

Over two-fifths (41%) of the total energy intake of diets reported by Aboriginal and Torres Strait Islander people in the National Health Survey 2011–2013 comes from “discretionary” food and drinks [19]. These are defined in the Australian Dietary Guidelines (ADGs) as food and drinks that “are not a necessary part of a healthy diet, and are high in saturated fat, added sugars, salt and/or alcohol” [20].

A lack of food security—when all people have “physical and economic access to sufficient, safe and nutritious food to meet their dietary needs and food preferences for an active and healthy life” [21]—is also problematic for Aboriginal and Torres Strait Islander Australians. In response to a question measuring (one aspect of) food security in the 2011–2013 National Health Survey more than one in five (22%) reported living in a household that, in the previous 12 months, had run out of food and had not been able to afford to buy more [19]. This proportion was higher (31%) among respondents in remote areas [19]; however, most (70%) obtained food from other sources [19], testament to community resourcefulness and resiliency, and the “safety net” provided by the cultural norm of sharing [17].

With a few exceptions, efforts to improve food security and nutrition of Aboriginal people living in remote communities have been fragmented, largely ineffective, but rarely published [3,22,23,24]. One common feature of successful programs is leadership by the communities involved [24,25]. Much of this work has focused on improving food supply in local stores [26]. For example, the communities on the Anangu Pitjantjatjara Yankunytjatjara (APY) Lands in South Australia have increased availability and affordability of healthy foods, including fruits and vegetables, lean meats, and wholegrain cereals, which share some of the protective properties of traditional foods [3]. However, despite concerted effort over three decades, dietary intakes at community level had not improved substantially [3]. Hence, there has been growing interest in exploring factors driving food choices at household level, seeking more granular data to inform a more targeted response [3].

However, investigation of household food practices in the local social and economic environment [18] and of Aboriginal perspectives on food choice has been limited [2,17,27]. This is in part due to methodological challenges in investigating and capturing what Aboriginal people are eating [28,29]. Direct questioning and noting individual behaviour, such as food consumption, is traditionally considered impolite [28]. Obtaining a complete record of foods consumed is also challenged by selective recall and under-estimating consumption of certain foods as participants may offer the right information according to perceived social desirability [30], rather than a technically accurate response [28,31,32].

### Study Conceptualization and Aims

The Ngaanyatjarra Pitjantjatjara Yankunytjatjara (NPY) Women’s Council, and our Child Nutrition Program staff (particularly S.B.), conceptualized and implemented this study in an effort to explore what Anangu (the local Aboriginal people on the APY Lands) were “really” eating and why. Importantly, the NPY Women’s Council recognised the study required novel, pragmatic, and sensitive approaches in order to “meet people where they are” and obtain honest and comprehensive information about household food practices. The study was entitled *Maitjara Wangkanyi*, a Pitjantjatjara phrase, which translates to “talking about food”.

The study aims were to investigate:

1. What food and drinks Anangu families are eating; and
2. Factors that influence these food choices.

## 2. Materials and Methods

### 2.1. The Setting

**Figure 1:**
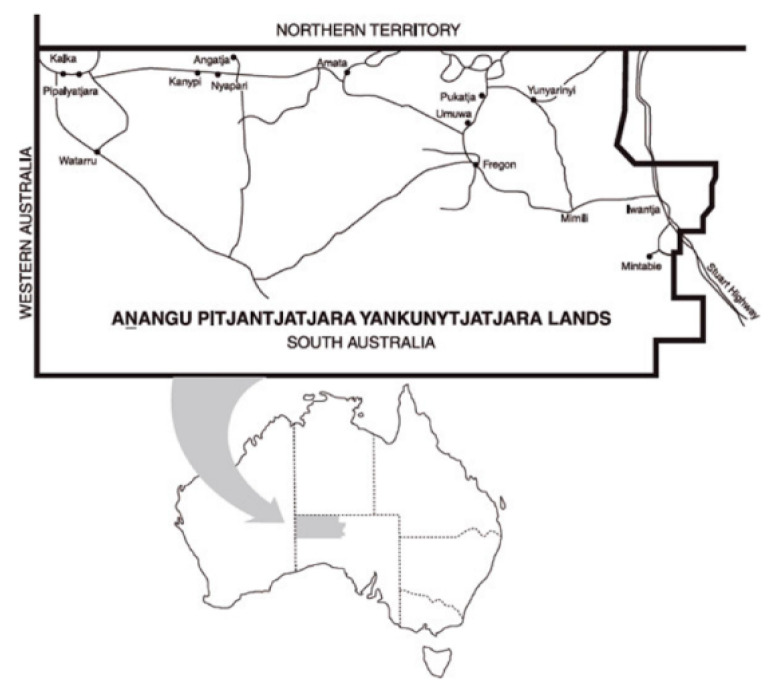
Map showing the Anangu Pitjantjatjara Yankunytjatjara (APY) Lands.

The NPY Women’s Council (established in 1980) is led by women’s law, authority, and culture to deliver health, social and cultural services for all Anangu across 26 desert communities in the tristate region of Western Australia, South Australia, and the Northern Territory. These include the communities of the APY Lands, a remote area covering about 105,000 km^2^ in South Australia, 40 km south of the Northern Territory border and around 250 km west of the Stuart Highway (Figure 1). The APY Lands, on which this study was conducted, is home to around 510 families and 2280 people, about 1900 of whom are Anangu [33]. The Pitjantjatjara Land Rights Act 1981 provides inalienable freehold title. Anangu live in an adaptive traditional way, and Pitjantjatjara and Yankunytjatjara are the first languages. The median equivalised household income on the APY Lands was AU $459 per week in 2016, around half the corresponding national figure (AU $877 per week) [34]. Public health services provided by the Nganampa Health Council (NHC), an Aboriginal community-controlled health service organization, include initiatives aimed at improving nutrition and wider food security through the Uwankara Palyanyku Kanyintjaku (UPK) program [35]. The Child Nutrition Program of the NPY Women’s Council has provided community nutrition promotion and family-focused support, especially for children with growth faltering, for 24 years [36].

### 2.2. Study Approach

All elements of this study were guided by a commitment to culturally-appropriate and ethical research [37]. Recognising that Australia’s Aboriginal peoples have been “over-researched” [38,39] and that among Anangu, eating is a private activity, it was carefully considered how participants would be approached and engaged in the research. Ethnographic [40] and Indigenist [41,42] research methods were applied in ways that would ensure participants’ comfort and encourage openness [43,44]. In the international context, Indigenist research methodologies respect and prioritize Indigenous peoples’ ways of knowing, being and doing by using methods that centre Indigenous peoples in the research process in a way that is respectful, recognizes their needs and interests and place their voices at the centre of the research process [41,42,45].

The study design was endorsed at the annual general meeting of the NPY Women’s Council in November 2013, attended by about 150 women from the APY Lands, the Northern Territory and the Ngaanyatjarra communities in Western Australia.

### 2.3. Ethics and Privacy

Ethics approval for this study was obtained from the Aboriginal Health Council of South Australia Incorporated (reference number 04-14-573). After a verbal explanation of the purpose of the study and research methods, participants were given an information sheet and consent form (available in Pitjantjatjara and English), and assured information provided would be confidential. To ensure participants’ anonymity and privacy all identifying information has been removed. We also delayed publication of data to allow for geographical movements and changing configurations of families over time that would reduce the potential for families to be recognised.

### 2.4. Positionality of the Researchers

Consistent with ethnographic and Indigenist methodologies, it is crucial for the researchers to locate themselves within the research [46]. The first author (S.B.) has lived and worked with Anangu in Central Australia for more than 40 years and has worked as a Senior Child Nutrition Development Officer with the NPY Women’s Council for the past decade. S.B. is proficient in Pitjantjatjara and led the data collection and interpretation. I.S. is a Pitjantjatjara woman who was born on the APY Lands and has lived there most of her life and is S.B.’s niece by marriage. I.S. and S.B. worked together to ensure informed consent and accurate data collection. Both have kinship connections and cultural legitimacy in the communities in the study setting. As community members and cultural ‘insiders’ [47], S.B. and I.S. had pre-existing knowledge of the research and community context and “expediency of access” to the field [48]. Their ongoing relationships with families in the community engendered trustworthiness, and also imposed a sense of “relational accountability” [44].

A.L. has ongoing connections with people on the APY Lands since living and working there in the early 1980s and was an advisor regarding study design and methodology; L.-M.H., B.W., and M.L. became involved in the study at the point of data analysis, interpretation, or write up. Throughout the process of data analysis and producing this manuscript, these authors—who are neither Aboriginal nor community members—foregrounded reflexivity in an effort to support and work alongside the community-based researchers [49,50]. S.B. had most intimacy and embodied knowledge of the data and guided representation and interpretation.

### 2.5. Study Participants

Data were collected from volunteer members of households in one of the larger homelands (community “A”), one of the smaller communities (“B”), and one large community (“C”). There was no minimum or maximum number of participants; the plan was to recruit six to eight households and spend around seven days collecting data with the members of each.

Community C is the largest of the three communities, with around 110 households, and community A the smallest with around 20 households [33]. There is a local food and general store in communities B and C; community B’s store is in relatively close proximity to community A. In 2016 the median weekly household income of Aboriginal and/or Torres Strait Islander households was $933 in community A, $1125 in community C, and $1562 in community B [51]. Access to the communities is by small aeroplane or road, with weekly truck supply of fresh, frozen and dry groceries and other freight. There is a twice weekly “bush bus” for personal travel from the communities to Alice Springs and return. The communities are ”dry”, in that alcohol is prohibited [52].

Recruitment was opportunistic, via face-to-face interactions and conversations in the selected communities, including informal meetings and talking to people outside the community stores about the study. Due to high rates of movement of Anangu between communities, and between houses within communities, data were collected for households, rather than individuals or families. A household was defined as one or more family groups that lived in one house. Each household was coded (Table 1) with a letter representing the community in which they lived, and a number. Household composition was recorded as the number of adults (females and males) and children (gender not recorded to reduce potential for identification of families) belonging in the household, including any long-term visitors, at the start of the data collection period, and changes during the period were recorded. Adults were coded according to the first letter of their gender, and all household members were numbered arbitrarily.

### 2.6. Data Collection

Data were collected by S.B. and I.S. in July and August 2014. They visited each participating household, the food stores and the communities, in order to observe food shopping and preparation/cooking—as well as family life, household infrastructure, and social and cultural events—and engage in food-related conversations. An open-ended and conversational approach gave participants greater control over disclosure, consistent with cultural norms, and facilitated richer data than more formal research processes would [43]. Conversational methods are also congruent with Indigenous paradigms, being grounded in relationality and Indigenous ways of transmitting knowledge through storytelling and “yarning” [43,53].

At a household level a range of contextual and dietary data were collected: income (amounts, when received, and the family group with whom income was shared); reported food and drinks consumed; and foods and drinks purchased. During each household visit, one or more adults reported all foods and drinks consumed by household members in the preceding 24 h. There was minimal questioning or use of probes, and no attempt to estimate portion sizes. S.B. and I.S. also noted information provided about when, where and how meals and snacks were prepared and eaten, and how many household members and/or visitors consumed meals. In addition, they collected shopping dockets/receipts for as many shopping trips by participating household members during the data collection period as possible.

Detailed field notes were recorded (in English) in individual record books for each household. Codes were used to identify house occupants, and participants were assured each book was “secret” and that no identifying details would be reported. In addition to field notes in individual household record books, S.B. and I.S. recorded data from observations and conversations and reflections in research diaries and reports. The observations and comments included in Table 1 reflect the style of entries in these sources.

### 2.7. Data Analysis

Congruent with ethnographic and Indigenist methodologies, data analysis was largely inductive [54]. The research aims framed data sorting, analysis and reporting. S.B. and L.-M.H. extracted descriptive information about each household, including usual composition, number of days observed, estimated weekly income and the presence of food storage, preparation, and cooking facilities/equipment, as well as the number of “hungry days”—on which two or more meals were missed or only minimal food (e.g., bread and tea or *arngu* (flour gruel)) was consumed—from the individual household record books to Excel spreadsheets. Details of food and beverages purchased, including brand and size, were extracted from the shopping receipts to spreadsheets for analysis by household. Items were classified by food group, as defined in the ADGs [20], and energy and macronutrient content analysed using FoodWorks7 Professional software (Xyris Pty Ltd., Brisbane, Australia) [55].

In late 2019, S.B. contextualized the recorded data in conversations with A.L., L.-M.H., and B.W., sharing insights and preliminary connections from observations and conversations she had noted during data collection. L.-M.H. audio-recorded and transcribed, or made written notes of, S.B.’s narratives. All qualitative data, including field notes in household record books, research diaries, transcripts and narratives, were collated, synthesized and thematically analysed to identify concepts and patterns, which were then discussed with the other authors.

## 3. Results

Before presenting the qualitative ethnographic data, results are presented in the order of household characteristics and income, expenditure on food and drinks, and dietary data.

### 3.1. Household Composition and Income

As reported in Table 1, 13 households, with four to 13 occupants (more than 105 people in total), engaged in this study. The period of data collection with each household ranged from four to 11 days (Table 1) with a mean of seven days. In several households, variability (changes in the number of household members during the study period) was high (Table 1).

Participants received income from a variety of sources, including from artwork, but most were not in the paid labour force, or were unemployed and received government welfare. Most people receiving government welfare had chosen to have a proportion of their payments debited to a “BasicsCard”, an Australian Government income management initiative introduced in various locations including the APY Lands. Cardholders cannot withdraw cash or buy tobacco or alcohol (not sold on the APY Lands); the card can only be used to purchase essential items such as food and clothing. Household members generally pooled individual incomes. The approximate weekly total reported income for each household is reported in Table 1. The mean weekly reported household income was $1323 (sd $266) in community A, $1168 (sd $613) in community B, and $1389 (sd $664) in community C. In most households, one woman (sometimes two) managed most of the food purchasing and preparation.

### 3.2. Household Food and Drink Purchases

Estimated total expenditure on food and drinks by each household during the period of data collection ranged widely from $74.71 to $905.90, with the median being $262.53 per household per week and the mean being $350.19 per household per week (Table 1). According to analysis of the shopping dockets, on average, nearly half (46%) of household expenditure on food and drinks was on products categorized as “discretionary” in the ADGs [20] (Table 2), commonly sugar-sweetened beverages (SSBs); takeaway foods such as pies and pizza; confectionary; biscuits and potato crisps. Households spent a mean of 18% of their food expenditure on meat, poultry, eggs, and/or other alternatives; 13% on grain (cereal) foods; 7% on milk, yoghurt, or cheese; 9% on vegetables and legumes/beans; and 5% on fruit (Table 2). However, across the households there were large variations in spending on different food categories. The proportion of the household food budget spent on meat, for example, ranged from 3% in C6 to 36% in C5, and the proportion spent on discretionary food and drinks ranged from 72% in C2 to 21% in C5 (Table 2).

### 3.3. Dietary Intake

Estimated consumption of each food group by the household’s usual occupants (adults and children) over a fortnight is presented in Table 3. On average almost half (46%) of the total energy intake of household members came from discretionary food and drinks. A further 32% of energy intake was derived from cereal foods. In most households (9 of the 13) more than 75% of total energy intake came from these two food categories. Meat and other protein sources comprised 10% of energy intake on average; milk, cheese, and yoghurt contributed 6%; and vegetables and fruit together contributed 6% (Table 3).

Analysis of each household’s energy intake by food categories (Table 3) identified three main dietary patterns in households: one similar to the mean; the second very high in discretionary food and drinks; and the third high in cereal foods but with lower than average intake of discretionary food and drinks. For example, household C7 was typical of the mean consumption pattern, with dietary intake including a mix of staples like bread, flour, and Weet-Bix^TM^; milk powder and eggs; smaller quantities of meat and milk; and low intakes of vegetables and fruit. The second dietary pattern was seen in households C2 and A2, with 65% and 62% of total energy intake respectively derived from discretionary food and drinks, including SSBs, confectionary, takeaway foods, frozen pizza and party pies. The third dietary pattern was exemplified by households B1 and C5, which had the lowest proportion of energy intake from discretionary food and drinks across all households (25% and 12% respectively) and also the highest proportions of energy from grain (cereal) foods (58% and 57%, respectively). In addition to bread, damper (a type of leavened bread usually made from flour, water and baking powder, cooked in the coals of a fire) and Weet-Bix^TM^, the diet of household C5 was high in oats and rice, the latter not commonly consumed by other participants.

### 3.4. Eating Patterns Reported by Anangu

It was unusual for household members to have three meals each day; most commonly they had only two. From the participants’ recall of food and drinks consumed the previous day it was clear that hungry days were very common. In nearly all households people described one hungry day in a week, with several households reporting two hungry days a week (Table 1). On other days when the household did not have available money or food, participants reported obtaining something to eat from other families, or sourcing meals elsewhere e.g., attending events at a school, community centre or workplace at which food was provided. Children generally had lunch at school on weekdays. In several households, aged residents or those with a disability received a Home and Community Care (HACC)-provided lunch each day.

Breakfast was the most regular, and often most substantial, meal consumed daily. Packaged cereals (largely Weet-Bix^TM^) and bread were commonly reported breakfast options, as were eggs with toast and tinned spaghetti, baked beans or bacon. Dinner or lunch generally included meat, commonly lamp chops, mince, kangaroo tail or chicken. Meals cooked at home were largely dominated by grilled, roasted or tinned meat, with bread, damper or noodles, and sometimes vegetables. Reported ‘main meals’ also commonly included takeaway foods, such as meat pies, pizza, and ham and cheese croissants that were heated in the store and consumed near there. Weet-Bix^TM^, bread and instant noodles were common staples in many households, eaten at any time of the day, particularly by children.

There was limited intake of traditional bush foods. Only two households reported members going hunting during the study period and both were unsuccessful in finding large animals; one gathered *tjala* (honey ants) and the other *maku* (witchety grubs). Some participants reported eating and/or were observed making damper, but the majority bought bread from the store.

### 3.5. Factors Influencing Food and Drink Choices

Several key factors influenced household food choices and practices. Overall household composition and organization, particularly the number and mobility of household members, clearly affected the purchasing, availability and preparation of food. Overcrowding, with many people, particularly children, sharing household facilities, was a hindrance to preparing or sharing meals. Children also influenced food purchasing directly via their demands for confectionary and snack foods. A male adult in household C9 explained the only way he and his wife could do “good shopping” was to leave the children at home so they could not “control the shopping”.

In A2, there were up to 15 adults, teenagers, and children in the household during the data collection period. Despite the household’s relatively high pooled income and buying power, the families did not cook more often, and experienced similar hungry days to families with less apparent purchasing power. In another household, C4, the matriarch reported walking into the bush and building a fire to roast a kangaroo tail and vegetables so that she could “cook in peace”; she carried the cooked meal back to share. In contrast, despite the number of occupants (13), more cooking and eating at home was observed in household C5, in which the two matriarchs had more structured shopping and eating routines than most other households and expressed interest in improving their diet. This household also had the lowest proportion of total food and drinks expenditure on (and energy intake from) discretionary items. Conversely, absence or illness of a household matriarch significantly impacted food intake; for example, in C1, when F1 was ill, there was no food prepared in the household.

Available income was a primary determinant of when, and how much, food was purchased and consumed. Only one participant described planning shopping rather than spending income immediately it was received: F1 in household C7 managed food for four adults and nine children in her household and reported saving money from her weekly wage paid on Thursday to do a “big shop” on Saturday. Mostly the household matriarch shopped for food on the day that a household member’s pay or benefit was received. It was common that within a few days the households had, as one participant stated, “no money, no *mai* [food]”. The reported food intake of household C4 is an example of this ”feast and famine” cycle: on the first day of data collection the adults recalled eating *arngu* or nothing; after a household member received income, they bought kangaroo tail and vegetables and family members ate takeaway at the shop; the following day they reported having only *arngu* again; then after more income was received members bought food including vegetables and cooked meals. One participant explained that putting aside food or money (budgeting) for later was seen as anti-social and not respected.

Often when the family members who received or controlled income were absent, other household members were left without means to buy food. Conversely, households reported how absent family members—in one, a grandmother who was having dialysis in Alice Springs, and in another, the mother of several children left in their Aunties’ care—left their BasicsCard to enable food to be purchased for children. Participants willingly shared information about household income and were interested in seeing their household economy set out on paper; several discussed the potential for better management of income by, for example, requesting weekly rather than fortnightly pay.

Importantly, most of the households had limited resources and equipment for food storage, preparation and cooking. Availability (and functionality) of equipment observed during household visits is noted in Table 1. In several households there was no refrigerator or one that did not work. In a few houses there was a working refrigerator but it was empty. Participants in these households explained that food in a refrigerator or kitchen area would be visible and likely to be consumed immediately by household members or visitors, and that the absence of locks on doors and windows meant any food stored in the house could easily be accessed by kin or other community members while household members were away. Hence, having a small refrigerator in a bedroom, was a more common strategy to manage the food supply. In C1 for example, only bread was stored in the main fridge and only tea and sugar in the kitchen; meat and other food was kept in a fridge in the matriarch’s bedroom. In C7, the two women (caring for nine children) concealed most of the household food in their bedrooms and had requested locks be put on kitchen cupboards so they could be used for food storage. Another household reported not using available storage because of cockroaches. As a consequence, other than items such as tea, sugar, milk powder, and flour, most households did not buy food for consumption later in the week—food and drinks were mostly consumed on the day of purchase or the next day.

Most houses did not have a working oven. Much of the cooking that was observed or reported by participants was done on a stovetop or in an electric fry pan, and kangaroo tails and damper were cooked in fires or on coals outside. The resourcefulness of several of the matriarchs was demonstrated by cooking family meals in a single small saucepan. Household B2 was the best resourced in terms of cooking equipment, including a stove, microwave oven, deep fryer, wok and electric flat grill (Table 1). This was reflected in the household’s eating patterns, with members reporting several meals cooked at home, such as chicken pieces with salad and home-fried chips. During the data collection period several participants bought cookware and dinnerware, as noted in Table 1, indicating an interest in preparing and eating more meals at home. However, when the matriarch of C9 purchased cooking equipment, her husband insisted she return it to the store and buy more food instead.

Results suggested a tendency to choose foods and cook meals that were familiar, but observers noted that at community events where food was provided, participants were willing to try—and enjoyed—various unfamiliar foods and meals. In the course of the study, two participants also asked for advice about increasing variety in their diets or losing weight. For example, I.S. was in the store when a participant from C8 was shopping; they talked about food, and the participant bought fresh vegetables and a curry pack and asked I.S. to her home to cook with her.

## 4. Discussion

This study has highlighted the strength of Anangu resourcefulness and resilience in securing household food intake in the face of poverty and adversity. Collating information from observations, conversations and records, and ensuring analysis was data close (focused on determining patterns and themes in the data), has provided unique insights. These included the specific food and drinks people in the remote communities were buying and eating, the varied range of household dietary patterns and more nuanced insights into factors influencing food practices and food choices.

### 4.1. Study Strengths and Limitations

Strengths of this study’s design include the application of a combination of methods grounded in ethnographic and Indigenist methodologies to ensure cultural safety for participants [43], collect rich data and illuminate sociocultural factors that influence dietary beliefs and practices [56,57]. As community members (insiders) fluent in Pitjantjatjara and attuned to cultural protocol and practice S.B. and I.S. were granted access to households and able to observe and talk about practices that participants would be unwilling to discuss with “outsiders”. Our analysis of data from household shopping dockets confirmed that some participants initially did not recall all of the snacks, drinks and takeaway food they purchased (and usually consumed) at the store when reporting their previous day’s food intake. Editing of recall related to perceived social and/or nutritional desirability [30] has been identified in other research in Australian Aboriginal and Torres Strait Islander communities [58,59]. Having community-based researchers/authors (S.B. and I.S.) who were part of the community and, as S.B. explained, “hanging around at the store”, enabled collection of additional shopping dockets and observations that provided a more complete picture of food and drink purchasing patterns and dietary intake of people in the study communities. As a result, there was much higher concordance between methods than observed in past attempts to record dietary intake compared with store data [28]. The social capital of the APY Lands communities and support of the NPY Women’s Council were also fundamental to the feasibility and success of the study, helping engender a high level of trust between all involved.

There were some deviations from initial study design related to recruitment challenges and mobility of household members. Some people refused to participate in the study because they had, as one person explained, “things to keep private”. The original intention was to visit up to eight households to collect data on seven consecutive days, but family mobility and fluctuating interest in participation necessitated a more flexible and opportunistic approach. However, we found capturing snapshots of a few days, or information from non-consecutive days over a longer period (up to 14 days) from a larger number of households of different composition provided rich data.

There were a couple of unplanned interactions and activities during the study, as researchers responded to participants’ requests for information about specific foods, diets or cooking. Rather than refusing these requests out of concerns about contaminating data, researchers respected participants’ trust and keenness to try new things and provided appropriate advice. Given this very small number and limited reach, these events were not considered to have unduly influenced results.

### 4.2. What Anangu Were Eating and Why

Collating information about what Anangu participants reported eating (from the household record books) and what they purchased (from shopping dockets) enabled identification of dietary patterns and variations in types of specific food and drinks. As food intake was recorded at household, rather than individual level, and the composition of households varied, it was more meaningful to analyse and compare dietary intake qualitatively, rather than quantitatively. The calculated contribution of discretionary food and drinks to household energy intake in this study (46%) was higher than that reported (41%) in the national Aboriginal and Torres Strait Islander health survey [60], even though the latter also included alcohol, which is not available in the APY Lands. This could reflect the use of objective data in this study. Analyses of purchases and consumption showed large variation in patterns of household dietary intake, with extremes being relatively high in less expensive cereal (grain) foods such as bread and breakfast cereal, or high in discretionary food and drinks), providing valuable insights into the economics of food choice theory [61]. In most (9/13) households, more than 75% of total energy intake came from the combination of discretionary food and drinks and cereal (grain) foods. This had changed markedly from the 1980s, when results of store turnover studies found that meat, white sugar and flour alone provided over 60% of the energy intake in these same communities [62]. The dietary patterns identified were even further from traditional meat-orientated diets [5,63] than identified previously on the APY Lands in 1986–1987 and 2012 [3,28,62].

During the past four decades the price of meat, and the range and affordability of discretionary food and drinks, had increased dramatically in these communities [3]. These factors are likely to have contributed to the dietary changes observed, and the current dietary patterns are characteristic of suboptimal diets associated with poverty [61,64]. Notably, the two households in 2014 with the lowest proportion of discretionary food and drinks intake also had the highest intakes of cereal (grain) foods. The matriarchs in these households appear to be trying to limit intake of discretionary food and drinks—potentially because of health and/or cost concerns—and instead chose relatively inexpensive cereal foods, particularly bread, Weet-Bix^TM^ and oat porridge, to “fill up” family members, particularly children [61,65,66].

Collecting and analysing data at a household level highlights different food purchasing strategies, influences and the inappropriateness of assuming homogeneity of food practices or dietary intake at a community level. Findings reveal the variability in food choices between households, reflecting the diversity of household circumstances and resources including the number and mobility of household members; income (amount, regularity and management); availability of facilities/equipment for food storage, preparation and cooking; as well as individual preferences and food sourcing and purchasing strategies. S.B.’s and IS’s previous observations in the course of their work of significant variability in dietary intake between families was a driver for this study, as they felt community-level data were masking different coping strategies and food choices. As I.S. reflected during the study: “when you start looking, going deeper, asking why they got no *mai* at home [you find] all the different difficulties and blockages. There is this whole situation around money and buying food and it is like there is no answer so everywhere you look, ‘Oh, it’s for this reason’, but then there is another reason, it’s like build up, build up”.

### 4.3. Traditional Foods

Anangu speak of our own foods as *kuka* (animal foods), *mai* (plant foods), *maku* (edible grubs), and *tjuratja* (sweet foods) [67]. The ADGs for Aboriginal and Torres Strait Islander Australians [20] are: eat traditional bush foods whenever possible, and choose store foods which are most like bush foods. While limited intake of traditional bush foods was observed during the periods of study, Anangu reported *kuka*–*malu* (kangaroo), *kalaya* (emu), *kiparra* (bush turkey), *ngintaka* (perentie), and *tinka* (goanna)–being brought into communities regularly on a seasonal basis. An attempt was made to classify, synthesize and analyse foods and drinks purchased and consumed in this study into traditional groupings. However, it was not always clear where mixed and processed foods were best placed, and so these efforts were abandoned. Much more work is required to better accommodate Anangu perspectives into food security and nutrition programs.

### 4.4. Factors Affecting Dietary Choice: Availability and Convenience

The continuing high intake of discretionary food and drinks is in part driven by the large range and variety stocked in the community stores [3], including SSBs kept appealing cold in display fridges, and the convenience of pre-cooked foods in bain-maries and/or microwave ovens for customer use. I.S. noted that while Anangu know most take-away store meals are unhealthy, the fact they do not need storage, preparation or cooking made them popular: “It’s an easy meal, straight in, buy it, put it in the microwave and it’s done”. Popular staple cereal foods, such as bread, Weet-Bix^TM^, and oats tend to be available in the stores at all times too.

Both the meals reported by participants, and products purchased according to shopping dockets, also reinforce the role of familiarity and convenience in food choice [13,68]. Continuing preferences for white bread, meat (fresh and tinned) and foods high in sugar have been attributed to colonial history, childhood diet and satisfaction or feeling “filled up” [16,18,27,62,69,70,71]. S.B. noted community members’ preference for “full belly” satisfaction, which was commonly met by bread or damper and “cool drinks” (SSBs). Further, these foods require little or no equipment to prepare and cook.

We found most houses lacked adequate facilities to store and prepare food and cook meals, such as storage space, preparation bench, a working oven and fridge, as identified in earlier studies [11,35,70]. For some families, not having a fridge, and fear of cockroaches walking over their food, meant they could only buy food for the day. I.S. noted that several participants had the ability, and desire, to cook healthy meals if they had “the money and the right resources–the pots and pans”. As a consequence of the study design, some participants showed interest in increasing familiarity and practical experience with different foods, overcoming an identified barrier to cooking [16].

Overall, however, this study provides further evidence of the heavy reliance on take-away and convenience meals and declining cooking by Anangu families, consistent with changes seen in Australia more broadly [3]. This also reflects taste preferences for fat and sugar and a desire to consume food immediately documented in Aboriginal groups in Central Australian communities [5,18,72], and adds to the literature about taste socialization through family and community interactions [72]. Many households were feeding extra visitors, or were commonly approached by other families seeking food, so members may have chosen to buy takeaway and eat at the store to avoid cultural norms of reciprocity and generosity [18,73]. Given that participants’ shopping trips often followed a hungry day, immediately satisfying hunger is likely to be a driving factor.

### 4.5. Factors Affecting Dietary Choice: Income

In Australia, the poverty line (defined as 50% of median income) is $457 per week for a single adult [74]. At $459, the median equivalised household income on the APY Lands was only 52% of the corresponding national figure in 2016 [34], highlighting the magnitude of deprivation in this region. The mean weekly reported household income in the communities in this study was $1344 (sd $599), with households accommodating a mean of eight people, as detailed in Table 1. This is similar to the median weekly household income, for around half this number of people, reported in the 2016 Census for all people on the APY Lands ($1150) [51]. This was 50% more than reported in the 2016 Census by Aboriginal and/or Torres Strait Islander households (mean 4.7 people) on the APY Lands ($893), but 30% less than reported for non-Indigenous households (mean 3.8 people) on the APY Lands ($1914) [51]. These comparisons highlight the challenges in documenting sensitive, varied and sub-optimal income data at household level. Importantly, however, all available data confirms that Aboriginal incomes on the APY Lands are much less than corresponding figures for Australia as a whole [34]. Yet food prices in these remote communities are up to 50% higher than in urban centres [1]. These data highlight the critical role poverty plays influencing food choice in these communities.

For many households the income/welfare benefit payment schedule still determines when, and how much, food can be purchased. The concept of feast and famine periods or fluctuating food purchasing behaviour related to the cycle of welfare/income payments has been well-documented [58,70,71,75]. However, across the households the incomes of family members were fairly dispersed over a fortnight and hungry days were not as predictable as expected. People receiving regular income were as vulnerable to hungry days as those reliant on government assistance. Hungry days did not only occur in the “off week” (for those receiving fortnightly income), but often the day or a few days after income was received, due to the pressure to purchase (and consume) food immediately after receipt of income rather than plan meals and budget.

Having “insiders” [47] collecting data meant participants were more comfortable reporting household income and sharing strategies for maximizing resources available for food purchasing. Many participants—like the majority of Aboriginal population living in very remote communities [76]—were unemployed or not in the labour force and their only form of income was government benefits. Of these, most used a BasicsCard, a cashless debit card with a quarantined proportion of welfare payments (usually 50% but may be up to 80%) that cannot be used to withdraw cash or purchase alcohol or gambling products. Under legislation only the holder of the card can use it. However, this study highlighted participants’ resourcefulness in pooling income and the importance of being able to access income locked to a BasicsCard in several households in which adults were the primary carers of their nieces, nephews, grandchildren or other children whose parent was absent due to illness, work or other reasons. Having a BasicsCard helped families with budgeting. I.S. observed that families that previously were “mai wiyatjara nyinanyi” (living with no food) now had a more consistent supply of food.

Findings also highlight the social capital in remote Aboriginal communities and the importance of school meal programs and community events in terms of food security. Despite the prevalence of hungry days across participating households, resilience to greater food insecurity was enabled by the culture of demand sharing. When households were without food and the means to buy more, many were able to obtain either food or money from family, neighbours, or government/community assistance schemes.

### 4.6. Factors Affecting Dietary Choice: Household Composition and Organization

In addition to impacting the household economy, household composition, and organization influenced dietary intake through impacts on eating patterns and preparation of food. Household size in Aboriginal communities is driven by kinship connections and a culture of sharing accommodation [77]. A household’s composition is highly variable, fluctuating due to visitors (usually extended family members) coming and going for various reasons including eviction from elsewhere, seeking refuge from violence or other problems, or temporarily visiting a community for *sorry business* (after a death) or work, for example [78]. Overcrowding is common—in one in four homes (24.6%) in remote and very remote Australian Aboriginal and Torres Strait Islander communities—and puts stress on infrastructure including food preparation areas and cooking facilities [78,79]. The homes of most participants had inadequate space and facilities for the number of occupants, and the chaos of overcrowded households—particularly those with large numbers of teenagers and children–clearly was not conducive to food preparation or cooking.

Despite the barriers, there were indications of the desire and willingness of participants to prepare and eat more meals at home. We observed some changes in food purchasing behaviour, as well as purchases of cookware and dinnerware. However, given the short period of observation and lack of follow-up it is not known whether efforts were maintained, were a Hawthorne effect [80], or were impacted by social or other circumstances. In addition to the example reported in the results of the participant whose husband insisted she return cookware she had bought and instead spend the money on food, another participant’s cookware purchased during the study period was later taken by others when she left her household due to a family crisis.

### 4.7. Implications

Many of the identified factors impacting food choice have been identified previously; however, the findings of this ethnographic study highlight the complexity and multiplicity of influences on household food practices. They reinforce what has been long known: that improving the dietary intake and nutritional status of Aboriginal people in remote communities requires fuller understanding of the social, cultural, historical, environmental, and economic factors that influence dietary intake, and that social action and practical support will be more effective than nutrition education [81].

Findings point to the need for further supply side initiatives to increase access to healthy, convenient, culturally-acceptable food in remote communities, including increasing availability of affordable healthy ready-to-eat meals and snacks (and reducing the availability and promotion of unhealthy alternatives); encouraging cooking through stocking a greater range of affordable cooking equipment in the stores (a central plank of the UPK program across the APY Lands [35]); and restricting availability of discretionary items, particularly SSBs. They also support the potential of community-led interventions to increase demand for healthier options, such as in-store and point-of-sale nutrition promotions and practical nutrition education activities, the success of which has been established subsequently in the APY Lands [82].

## 5. Conclusions

This study has highlighted the strength of Anangu resourcefulness and resiliency securing food in the face of poverty and many other adverse factors. The study has provided unique insights into the varied range of household dietary patterns, and more nuanced insights into a broad range of determinants influencing food practices and choices. These include household economic cycles and coping and budgeting strategies; overcrowding and family structures, mobility and ‘organization’; available resources (financial, and for food storage, preparation, and cooking); and familiarity and convenience.

The findings can help inform multi-strategy approaches to drive demand and help increase the availability, affordability and acceptability of healthy choices in community stores, including in-store and point-of-sale nutrition promotions, practical cooking sessions, and sharing local food-related knowledge. However, improving nutrition and health requires a genuine commitment to improving food security through reform that ensures Aboriginal and Torres Strait Islander people are the decision-makers to address the required structural and systemic changes.

## Figures and Tables

**Table 1:** Characteristics of participating households and observations of food practices.

Household	Days Observed (*n*)	Composition: Number of Females (F), Males (M), Children (C)	Variability in Household Size During Data Collection Period *	Approximate Household Weekly Income	Total Household Expenditure on Food and Drinks During Data Collection Period	Hungry Days (*n*)	Food Storage, Preparation and Cooking Equipment	Observations and Comments
A1	7	10: 5F 2M 3C	Low	$1350	$92.08	2	No fridge; several saucepans	On 5 nights, healthy meat and vegetable meals were reported. F1 described eating well during childhood and had learned to cook from her mother (who had run a community kitchen). Household included long-term visitors (1F and 2C).
A2	8	9: 3F 3M 3C	Medium	$1635	$197.67	2	Fridge (large, working, empty); stove not working; fry pan purchased during study	Very little cooking observed or reported; only 4 main meals (lunch or dinner) reported in 8 days. Up to 15 people in household—some family members sleeping outside in tents. Large vehicle available so much family movement between communities; 4 members were away for 4 days. Household members went hunting one day–no kangaroo, had only honey ants. F1 spent most of an emergency relief fund payment on lollies and cake “because the kids were crying”.
A3	7	4: 2F 2M	Low	$985	$74.71	2	Fridge (recently purchased); stove; fry pan; kettle; toaster; electric sandwich maker. No saucepans or cutlery.	In 7 days, 6 main meals reported, most comprising tinned meat, bread and/or noodles. A visiting daughter, who was learning to cook at trade school, prepared prawns and salad one night. F1 bought a high number of discretionary items, explaining it was to “please” her son with snacks and drinks he likes. F1 is diabetic and reported little food intake e.g., only a mandarin and banana one day. One night another household gave F1 food for dinner.
B1	6	6: 2F 2M 2C	Low	$555	$518.79	2	Hotplates; two small saucepans and a billycan (bought on day 1); bowls and cutlery and stockpot and additional chopping board (also bought during data collection). No fridge; not using kitchen cupboards for storage due to cockroaches.	Household included two couples, one with two children. The couple without children had insecure income and often did not eat with the other family (had at least two hungry days). For the couple with children, meat (fresh or tinned) with damper or bread was the most common meal. Vegetables (particularly pumpkin) reported in several meals (home-made stews). Two-minute noodles common on days when there was less money. Children ate lots of Weet-Bix^TM^. F1 purchased cooking and eating equipment during observation period. I.S. encouraged husband to join in with cooking; he asked for the recipe and reported later that they had repeated the meal.
B2	6	9: 4F 2M 3C	Low	$1780	$262.53	1	Fridge, stove, fry pan, electric flat grill, microwave, wok, deep fryer, sandwich maker	In 6 days observed, six dinner meals reported. Household reported eating a lot of chicken (bought frozen) and damper (one night had chicken with home-fried chips and salad pack), and vegetables on 3 days (included in stews). Children had breakfast and lunch at school, including fruit (bananas and oranges) and Weet-Bix^TM^. F1 reported driving to store 30km away for “cheaper shopping”.
C1	11	10: 3F 4M 3C	High	$2275	$604.71	1	Small fridge in main kitchen; concealed fridge in bedroom; oven and stovetop (working); cooking fire outside; pots	Household included one family of visitors (5A, 2C). Recalled lots of snacks of take-away foods e.g., pies, pizzas; and lots of tinned meat and Weet-Bix^TM^ reported. Household was gifted fresh bullock meat (two meals). When matriarch was sick on days 6 and 7 the family food supply was impacted (no food intake reported one day).
C2	8	4: 2F 1M 1C	High	$1460	$252.83	1	Stovetop Fridge broken	One female and one child stayed only two days. Matriarch (grandmother) reported a lot of meat and vegetables/salad and fruit–but that did not accord with shopping dockets, which indicated high purchase of discretionary food and drinks. Matriarch received supported meal (lunch), which was her only meal some days. When asked about other meals she explained there was “no mai [food]; it’s a drought here”.
C3	4	4: 2F 2M	Low	$570	No dockets collected	1	Fry pan, saucepan, toaster, freezer	Household had been using freezer as fridge and not eating food as it had “turned to stone” and was “old food”, not fresh. Household members went hunting; made a meal of *maku* (witchety grubs) and water.
C4	8	9: 4F 2M 3C	Medium	$1665	$352.22	2	(Not recorded)	Two female household members away much of the period. In 7 days, 4 dinners reported. Day one was hungry day–adults had *arngu* or nothing; after income received, they bought kangaroo tail and vegetables and family members ate takeaway at shop; the next day–only *arngu*; two days later when more income was received, they purchased food including vegetables, and reported home-made meals.
C5	8	13: 6F 3M 4C (1 baby)	High	$975	$162.98		Fridge (broken); 2 electric fry pans, 4 saucepans, 2 fry pans, kettle, toaster.	One family (couple and baby) went to Alice Springs (leaving another child); another family (1M, 1F, 3C) moved into the house. Over 8 days, household reported 7 dinners, 4 containing vegetables. No income or food on day 4, but the children ate at school and adults ate at the Arts Centre/office BBQ. Two matriarchs expressed interest in improving diet to lose weight.
C6	8	8: 3F 5M 1C (baby)	High	$1000	$141.08 58% kJ 65% $	1	(Not recorded)	Two family groups–some shared and some independent eating; 2 M visiting; one F away half the observation period. Five dinners reported, some vegetables. Good breakfasts (mostly porridge). One hungry day for most–only bread and biscuits reported.
C7	5	13: 2F 2M 9C	Medium	$2550	$359.64		Fridge; locked cupboard and small fridge in bedroom; kitchen cupboards.	Two matriarchs (sisters), both working in community and bringing in wages. Breakfasts and lunches reported for most days, but not much evidence of dinners. Food stored in bedroom to conceal from children and visitors. F1 (mother of 2 of the children, Aunty of 7) saves her pay from Thursday to do “big shop” on Saturday, and has access to two other family members’ BasicsCards to buy food for children. F2 makes damper every day when present. F2 was away for most of the period; F1 also away a whole day and overnight to buy tires and food in Alice. One family (1F, 1M + 3C)—moved to another household during observation period.
C8	4	4: 2F 2C	Low	$1490	$905.90		(Not recorded)	Purchased large quantities of meat (fresh and tinned). Two dinners of meat and vegetable curry reported. One day F1 spent $495 on shopping, including a whole box of bread; the next day she was upset that all the food purchased had been eaten or taken.
C9	5	6: 2F 1M 3C	Low	$520	$627.31		Fridge and freezer, frying pan, no saucepans, some plates and cutlery	Household members visited the store daily and on most days purchased takeaway meals they consumed there. Dockets show on one day that both women shopped, F1 (paternal grandmother, who had been a HAAC cook) bought fish and vegetables; F2 purchased only take-away food and confectionary. F1 asked for dietary advice as she was diabetic.

* Household variability: High = large degree of change; half of more of the occupants were short-term visitors, or were away for more than a day, during the data collection period; Medium = visits or absence of less than half the usual occupants during the period; Low = no or little variation in household composition during the data collection period.

**Table 2:** Household expenditure on food and drinks (by Australian Dietary Guidelines [ADG] categories)—amount ($) and proportion of total spend (%).

Food Category (Per ADG Food Groupings)	Households																	
	A1	A2	A3	B1	B2	C1	C2	C4	C5	C6	C7	C8	C9	Mean	SD	Median	Q1	Q3
**Grain (Cereal) Foods (Mostly Wholegrain)**	$8.91	$9.85	$14.10	$106.10	$38.61	$68.55	$24.05	$31.86	$38.91	$12.14	$39.48	$80.87	$146.51	$47.69	$41.91	$38.61	$14.10	$68.55
	(10%)	(5%)	(19%)	(20%)	(15%)	(11%)	(10%)	(9%)	(24%)	(9%)	(11%)	(9%)	(23%)	(13%)	(6%)	(11%)	(9%)	(19%)
**Milk, Yoghurt, Cheese**	$16.21	-	-	$33.26	$12.07	$48.36	$14.54	$25.04	$2.19	$15.38	$47.56	$54.07	$29.50	$22.94	$18.60	$16.21	$12.07	$33.26
	(18%)	(0%)	(0%)	(6%)	(5%)	(8%)	(6%)	(7%)	(1%)	(11%)	(13%)	(6%)	(5%)	(7%)	(5%)	(6%)	(5%)	(8%)
**Fruit**	$5.92	$19.74	$7.96	$26.89	$12.65	$1.34	$0.95	$13.71	$4.37	$14.39	$16.73	$30.21	$35.81	$14.67	$11.05	$13.71	$5.92	$19.74
	(6%)	(10%)	(11%)	(5%)	(5%)	(0.1%)	(0.1%)	(4%)	(3%)	(10%)	(5%)	(3%)	(6%)	(5%)	(3%)	(5%)	(3%)	(6%)
**Vegetables and Legumes/beans**	$9.93	-	$5.91	$34.14	$26.30	$68.84	$13.83	$17.64	$25.36	$3.20	$76.02	$116.43	$65.52	$35.62	$35.37	$25.36	$9.93	$65.52
	(11%)	(0%)	(8%)	(7%)	(10%)	(11%)	(5%)	(5%)	(16%)	(2%)	(21%)	(13%)	(10%)	(9%)	(6%)	(10%)	(5%)	(0%)
**Lean Meats and Poultry, Fish, Eggs, etc.**	$21.80	$30.73	$15.16	$168.80	$31.33	$116.28	$18.42	$84.22	$58.14	$4.20	$14.14	$136.56	$105.01	$61.91	$54.41	$31.33	$18.42	$105.01
	(24%)	(16%)	(20%)	(33%)	(12%)	(19%)	(7%)	(24%)	(36%)	(3%)	(4%)	(15%)	(17%)	(18%)	(10%)	(17%)	(12%)	(24%)
**Healthy Oils and Spreads (Unsaturated)**	$5.33	-	$2.94	$5.88	$3.33	$30.57	-	$5.26	-	-	-	$39.47	$19.09	$8.61	$12.92	$3.33	-	$5.88
	(6%)	(0%)	(4%)	(1%)	(1%)	(5%)	(0%)	(1%)	(0%)	(0%)	(0%)	(4%)	(3%)	(2%)	(2%)	(1%)	(0%)	(4%)
**Discretionary Food and Drinks**	$23.98	$137.35	$28.64	$143.74	$138.23	$270.78	$181.04	$174.49	$34.01	$91.77	$165.72	$448.30	$225.87	$158.76	$114.72	$143.74	$91.77	$181.04
	(26%)	(69%)	(38%)	(28%)	(53%)	(45%)	(72%)	(50%)	(21%)	(65%)	(46%)	(49%)	(36%)	(46%)	(16%)	(46%)	(36%)	(53%)
**TOTAL**	$92.08	$197.67	$74.71	$518.79	$262.53	$604.71	$252.83	$352.22	$162.98	$141.08	$359.64	$905.90	$627.31	$350.19	$248.43	$262.53	$162.98	$518.79
**Number of Days of Data Collection**	7	8	7	6	6	11	8	8	8	8	5	4	5	7.0	1.9	7.0	5.3	8.0

**Table 3:** Proportion (%) of total energy intake consumed, by Australian Dietary Guidelines food group category, per household.

Food Category	Households																	
	A1	A2	A3	B1	B2	C1	C2	C4	C5	C6	C7	C8	C9	Mean	SD	Median	Q1	Q3
Grain (cereal) foods	19%	18%	45%	58%	31%	31%	20%	25%	57%	27%	28%	25%	36%	32%	13%	28%	25%	36%
Milk, yoghurt, cheese, and/or alternatives	10%	0%	0%	4%	5%	11%	7%	6%	2%	9%	13%	7%	6%	6%	4%	6%	4%	9%
Fruit	2%	5%	5%	1%	2%	0%	0%	1%	1%	5%	3%	1%	3%	2%	2%	2%	1%	3%
Vegetables and legumes/beans	3%	0%	6%	0%	4%	5%	3%	3%	14%	1%	2%	5%	5%	4%	4%	3%	2%	5%
Lean meats and poultry, fish, eggs, etc.	12%	14%	15%	10%	6%	11%	4%	10%	14%	1%	11%	10%	9%	10%	4%	10%	9%	12%
Healthy oils and spreads (unsaturated)	0%	0%	0%	0%	0%	0%	0%	0%	0%	0%	0%	0%	0%	0%	0%	0%	0%	0%
Discretionary food and drinks	54%	62%	31%	25%	52%	42%	65%	53%	12%	58%	44%	52%	42%	46%	15%	52%	42%	54%
